# Advancements in 2D and 3D In Vitro Models for Studying Neuromuscular Diseases

**DOI:** 10.3390/ijms242317006

**Published:** 2023-11-30

**Authors:** Haneul Kim, Gon Sup Kim, Sang-Hwan Hyun, Eunhye Kim

**Affiliations:** 1 Laboratory of Molecular Diagnostics and Cell Biology, College of Veterinary Medicine, Gyeongsang National University, Jinju 52828, Republic of Korea; skyk@gnu.ac.kr; 2 Research Institute of Life Science, College of Veterinary Medicine, Gyeongsang National University, Jinju 52828, Republic of Korea; gonskim@gnu.ac.kr; 3 Laboratory of Veterinary Embryology and Biotechnology, Veterinary Medical Center and College of Veterinary Medicine, Chungbuk National University, Cheongju 28644, Republic of Korea; shhyun@cbu.ac.kr; 4 Institute for Stem Cell & Regenerative Medicine, Chungbuk National University, Chengju 28644, Republic of Korea; 5 Graduate School of Veterinary Biosecurity and Protection, Chungbuk National University, Cheongju 28644, Republic of Korea

**Keywords:** organoid, neuromuscular junction, tissue engineering, neuromuscular disease

## Abstract

Neuromuscular diseases (NMDs) are a genetically or clinically heterogeneous group of diseases that involve injury or dysfunction of neuromuscular tissue components, including peripheral motor neurons, skeletal muscles, and neuromuscular junctions. To study NMDs and develop potential therapies, remarkable progress has been made in generating in vitro neuromuscular models using engineering approaches to recapitulate the complex physical and biochemical microenvironments of 3D human neuromuscular tissues. In this review, we discuss recent studies focusing on the development of in vitro co-culture models of human motor neurons and skeletal muscles, with the pros and cons of each approach. Furthermore, we explain how neuromuscular in vitro models recapitulate certain aspects of specific NMDs, including amyotrophic lateral sclerosis and muscular dystrophy. Research on neuromuscular organoids (NMO) will continue to co-develop to better mimic tissues in vivo and will provide a better understanding of the development of the neuromuscular tissue, mechanisms of NMD action, and tools applicable to preclinical studies, including drug screening and toxicity tests.

## 1. Introduction

Advances in stem cell culture have made it possible to generate three-dimensional (3D) self-organizing in vitro tissue models called organoids. Using stem cells that have the capability to self-renew and differentiate into many cell types [1], organoids mimic the microenvironment of real organs and can thus be used not only to model organ development and disease, but also for drug discovery and toxicity tests to replace animal experiments. Furthermore, organoids are expected to be applied in transplantation treatments, where personalized organoids can be generated using stem cells derived from the patient. Organoids have been developed for use in various human tissues, including the brain, gut, kidney, liver, retina, spinal cord, and pancreas [2,3,4,5,6,7,8,9]. However, modeling diseases in which multiple components affect each other, such as neuromuscular disorders (NMDs), remains a challenge. NMDs comprise a diverse group of rare conditions with a wide range of etiologies and complex pathophysiologies. They are caused by functional deficiencies of the nervous system, skeletal muscle (SkM), and neuromuscular junctions (NMJs). The NMJ is a specialized synapse between motor neurons (MNs) and skeletal muscle fibers with a complex signaling network [10,11]. Remarkable progress in understanding NMJ development and NMDs has been made by sacrificing experimental animals including non-mammals such as Drosophila [12], *Caenorhabditis elegans* [13], and zebrafish [14] as well as mammals such as mice, dogs, and pigs [15]. Specifically, mice are the most widely used animal model for NMDs but may not accurately reflect human NMDs because of anatomical and pathophysiological differences as well as molecular-level differences in the NMJ proteome [16]. Therefore, it is essential to create neuromuscular organoids (NMOs) that closely resemble human in vivo physiology as feasible, in order to overcome the limitations of animal models, ensure accuracy of drug–susceptibility testing, and provide effective treatment for NMDs [17,18]

Initially, the modeling of NMJs was performed by co-culturing SkM and MNs derived from induced pluripotent stem cells (iPSCs) under two-dimensional (2D) conditions, but it was found to be ineffective for clinical applications because it could not accurately replicate the in vivo 3D architecture and complex microenvironment of NMJs [19,20]. Compared to existing 2D co-culture systems, 3D NMOs enable not only long-term culture [21,22] but also the interaction between MNs and SkMs, as well as Schwann cells, which promote NMJ network formation and maturation [22,23]. Therefore, NMOs are generally considered a promising approach for modeling NMDs such as amyotrophic lateral sclerosis (ALS) and muscular dystrophy (MD).

**Figure 1:**
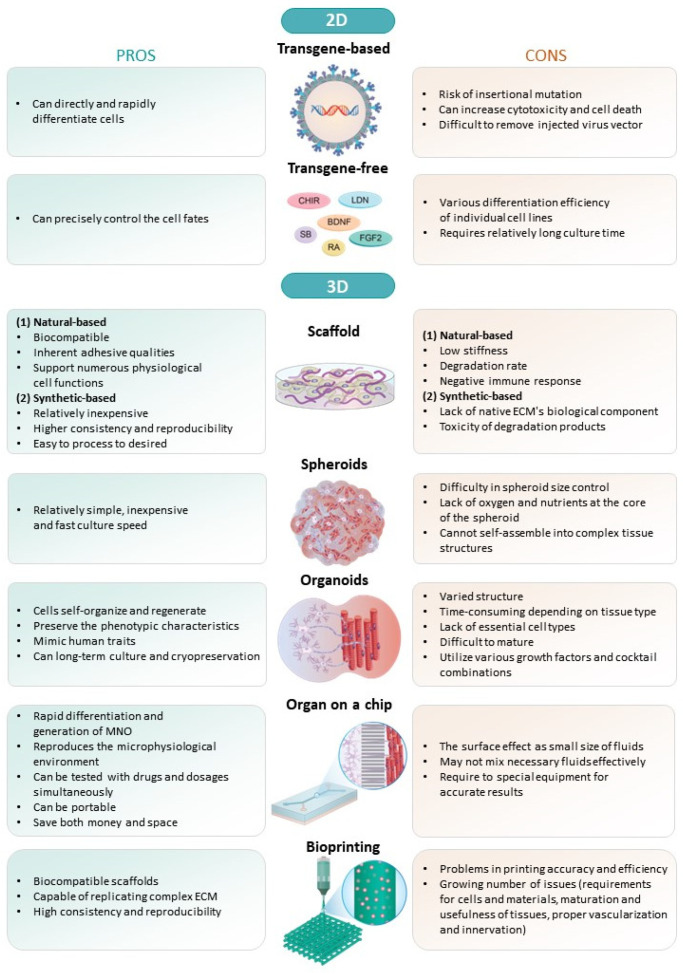
The pros and cons of 2D and 3D MN, SkM and NMJ models derived from hPSCs in vitro. MN, motor neurons; SkM, skeletal muscle; NMJ, neuromuscular junction; hPSCs, human pluripotent stem cells.

Studies have been conducted in several ways to develop in vitro models to reproduce the NMJ [17,24]. This review examines the pros and cons and the development of 2D and 3D models derived from embryonic stem cells (Figure 1) or induced pluripotent stem cells to study MNs, SkMs, and NMJs in vitro. We also discuss the application of the in vitro NMJ model for disease modeling and drug screening related to NMD.

## 2. 2D Culture In Vitro

2D culture has been widely used to develop in vitro NMJ models because they are inexpensive, user-friendly, and readily accessible for cell observation and measurement. The conventional 2D NMJ model has been used since 1967s to study the molecular mechanisms of NMJ formation, physiological function of NMJs, and pathology of neuromuscular diseases [25]; therefore, the model is well-established with ample literature available. There are two main stem cell-based methods for generating MNs and SkM cells in a 2D NMJ model (Table 1).

### 2.1. Transgene-Based Method

The expression of transcription factors (TFs) can directly and rapidly differentiate cells into specific cells, such as MNs or SkMs, using viral and transposon vectors. MNs can be induced by overexpressing TFs, such as master neuronal transcriptional regulator TFs (NGN2, ISL-1, or LHX3), in human embryonic stem cells (ESCs) and iPSCs [27,28,29,47,48]. Using a transgene-based method, human ESCs and iPSCs can be quickly differentiated into desired MNs by bypassing the neural progenitor stage. However, it can also generate insertional mutations that are randomly integrated into the host genome. To solve this problem, Goto et al. used a Sendai virus (Sev) that does not integrate with the host genome; however, the problem of virus removal after MN induction persisted [48]. SkMs can also be induced by the overexpression of key myogenic regulatory TFs (Pax3/7 or MyoD) and epigenetic modulator TFs (BAF60 or JMJD3) [30,31,32,49]. For optimization, a method was developed to remove intracellular SeV vectors using a temperature-sensitive SeV vector [31]. Another method has also been reported by other groups [27,50] and is based on non-viral mRNA-mediated reprogramming to mitigate the risk of insertional mutations [27,51]. However, it is cumbersome to synthesize mRNA, and transfected cells are more stressed because of the activation of the innate immune response against synthetic mRNA molecules, resulting in increased cytotoxicity and cell death. To avoid these risks, transgene-free small molecule-based cocktail methods have been developed.

### 2.2. Transgene-Free Small Molecule-Based Cocktail Method

Many previous studies have demonstrated that a combination of small molecules can replace all reprogramming factors [18]. The sequential addition of small molecules regulates specific cellular pathways and precisely controls cell fate, resulting in the highly efficient production of MNs or SkM cells from PSCs. To induce MN differentiation, various small molecules such as inhibitors of dual SMAD signaling, including Noggin, TGFβ inhibitors (SB431542), BMP inhibitors (LDN193189, DMH1, or dorsomorphin), and neurotrophic and growth factors including BDNF, GDNF, and NT3 have been used [36,38,39,51]. Various small molecules have also been used for SkM induction, such as GSK3β inhibitor (CHIR-99021), BMP inhibitors (LDN193189), HGF, and IGF1 [43,44,45]. 

However, this transgene-free small molecule-based cocktail method has some disadvantages: (1) it requires a relatively long culture time; for example, MN and SkM differentiation usually takes 4–6 weeks, and functional maturation takes a few more months, and (2) the differentiation efficiencies of individual cell lines vary even when using the same protocol [52]. 

Even if an optimized protocol is developed to overcome the problems mentioned above, it is difficult to mimic the real 3D NMJ microenvironment in 2D culture systems, such as cell–cell and cell–extracellular matrix (ECM) interactions. The limitations of this 2D culture system can adversely affect maturation [53]. To overcome this problem, a 3D culture system was developed.

## 3. 3D Culture In Vitro

Two types of 3D culture methods were used to form NMJs from stem cells (Table 2). These are (1) the scaffold-based platform and (2) the scaffold-free platform (organoid-like 3D cultures). The following sections review platform studies of spatial and temporal control of the microenvironment, such as organ-on-a-chip with microfluid and bioprinting [54].

### 3.1. Scaffold-Based

Scaffolds are porous, fibrous, or permeable biomaterials designed to mimic the microenvironment of specific tissues in a 3D space. When selecting scaffolds, it is crucial to weigh the advantages and disadvantages because the mechanical, chemical, and biological properties differ depending on the material used [68,69]. Scaffolds can be classified as either (1) natural or (2) synthetic scaffolds based on the type of polymer used. 

Natural based hydrogel scaffolds (collagen, fibrin, or Matrigel™) have been used broadly for NMJs, MNs, and SkMs [23,57,70]. Natural hydrogel scaffolds are biocompatible, possess inherent adhesive properties, and support numerous physiological cellular functions. However, disadvantages include low stiffness, a low degradation rate, and a negative immune response owing to the presence of impurities [71]. Li et al. investigated the growth response of human neuroblastoma cells in 2D collagen I, 3D collagen hydrogels, and Matrigel [72]. Neuroblastoma cells extended longer neurites in 3D collagen I. Compared with Matrigel, the collagen I hydrogel was more porous, fibrillar, and stiffer, which promoted neurite development and upregulated neurofilament gene expression. Therefore, neurite outgrowth and gene expression are influenced by the mechanical characteristics of culture matrices. Hinds et al. reported that skeletal muscle structure and function are affected by scaffolds (hydrogel-based collagen I/fibrin/Matrigel) and their concentrations [73]. By varying hydrogel matrix composition (i.e., cell–matrix interactions), Hinds et al. [73] showed the potential of greatly improving the contractile function of engineered muscle tissue, as evidenced by a 3-fold improvement in force amplitude between collagen I-based and optimized fibrin-based bundles.

Synthetic polymer-based hydrogels (polyester, polyethylene glycol (PEG), polyamide, and polylactic acid (PLA)) are relatively cheap and easy to process to achieve the desired stiffness or porosity, consistency, and reproducibility [74]. For example, Salimath et al. reported that arginine–glycine–aspartic acid (RGD) functionalization of synthetic PEG hydrogels facilitated the formation of multinucleated and differentiated myotubes by promoting cell adhesion, proliferation, and differentiation [75]. Polyvinyl alcohol (PVA) is used for low-protein adsorption covering cell culture plates to prevent cell substrate adhesion. Molyneaux et al. showed that consistently sized spheroids can be generated using multiple human glioma cell lines on PVA-coated cell culture plates [76]. Natural and synthetic polymers have been combined and used as composite scaffolds to improve the mechanical properties of natural polymer-based hydrogels. The disadvantages of synthetic hydrogels include a lack of native ECM biological components and the toxicity of the degradation products [74]. 

### 3.2. Scaffold-Free

Scaffold-free 3D cultures can be used to cultivate spheroids and organoids by allowing cells to self-aggregate. Spheroids are often used interchangeably with organoids. The two models offer platforms for mimicking in vitro tissues and organs but differ considerably in important aspects. Therefore, cells that are suitable for NMJ studies should be used [54].

#### 3.2.1. Spheroids

Spheroids are simple cell and free-floating aggregates cultured in spherical shapes. Spheroids can be cultured in scaffold-free conditions, so they are relatively simple, inexpensive, and have a fast culture speed [77,78]. 

Numerous studies have reported on NMJ models utilizing these spheroids. Li et al. investigated NMJ formation and functional recovery by injecting spheroids of neural crest stem cells (NCSCs) or human bone marrow mesenchymal stem cells (MSCs) into the gastrocnemius muscle of a rat model of denervation injury [79]. They observed that intramuscularly implanted NCSC spheroids could survive and engraft for up to 9 weeks compared to conventional single-cell suspensions. In addition, NCSC spheroids showed increased secretion of pro-regenerative growth factors involved in neuromuscular regeneration. To enhance maturation and create a cellular soma distinct from skeletal muscle tissues, Rimington et al. cultured 3D spheroids of iPSC-derived MN progenitors [62]. In addition, they provided a method for creating 3D bioengineered human neuromuscular motor unit tissues with physiologically functioning NMJs using MNs produced from iPSCs and primary SkM cells. Recently, an optimal workflow has been reported for developing an iPSC-derived MN spheroid model and analyzing its 3D structure in detail [55]. The regulation of synaptic transmission and neuronal function is largely dependent on the interactions between neurons and vascular cells. Osaki et al. investigated a co-culture model of MN and microvascular networks [80]. Human neural stem cell-derived MN spheroids containing glial cells and MNs demonstrated long-term stability and accelerated differentiation. Strong neuronal activity and neurite elongation were enhanced by co-culture with endothelial cells (ECs).

However, these spheroids have certain limitations. Spheroids cannot self-assemble into complex tissue structures like organoids [77]. Controlling spheroid size is also difficult in most scaffold-free approaches [81]. Spheroids (>500 µm) develop a multilayer structure and lack oxygen and nutrients in their cores. However, this problem can be solved by culturing using functional biomaterials. Nevertheless, the multilayer structure of spheroids is useful for tumor research [77,81].

#### 3.2.2. Organoids

Organoids are self-organized, complex, 3D multicellular structures derived from progenitor and stem cells (ESCs, iPSCs, and adult stem cells) in vitro. Therefore, organoids have cell types, shapes, and functions similar to those of in vivo organs and can accurately mimic human traits. Organoids can also be used for long-term culture and cryopreservation. These characteristics of organoids are used for modeling in various fields, such as disease research, precision medicine, and new drug development [69,77,78].

Faustino Martins et al. reported a self-organizing functional neuromuscular model that simultaneously generated spinal cord neurons, skeletal muscle cells, and terminal Schwann cells using hPSC-derived neuromesodermal progenitors [21]. Thus, these NMOs provide a platform for investigating the contribution of each cell type to the disease phenotype at various phases of NMJ development and maturation. Furthermore, Pereira et al. demonstrated that sensorimotor organoids with NMJs work physiologically and contain SkM, sensory and MNs, astrocytes, microglia, and vasculature [65]. Formation of neuro-mesodermal progenitors and subsequent organoids was similar to that reported by Faustino Martins et al. [21].

Organoids have several limitations. They may lack essential cell types, have varied structures, and may not mature easily. In the case of blood vessels, vascularization has been confirmed in the sensorimotor organoids model with NMJs developed by Pereira et al., but studies on their contribution to vascularization and their function have not yet been performed [65]. Recently, the co-development of vascular networks and the peripheral nervous system has been demonstrated in neuro-mesodermal assembloids, and functional maturation has been assessed along with the achievement of vascularization in organoids [82]. In the future, these models are expected to be used to investigate the impact of vascularization on the function of each cell type, such as neurons and muscle cells. In addition to these challenges, depending on the tissue type, the creation of a complex structure often takes 2–3 months. Various growth factor and cocktail combinations must be utilized at different stages of the process. Thus, organoid creation is expensive and time-consuming. Finally, these complicating factors for the creation of organoids may include a stringent reliance on growth factors and signaling gradients to ensure lineage specification and balanced stem cell renewal. This problem can be solved using microfluidic technology [69,78,83].

## 4. Hybrid

### 4.1. Microfluidics and Organ-on-a-Chip

Microfluidics is a branch of science-technology that uses channels ranging in size from tens to hundreds of microns to precisely manipulate and process microfluidic (10^−9^–10^−18^ L) fluids [84]. By incorporating this technology, organ-on-a-chip (OOC) refers to a microfluidic cell culture chip that mimics the physicochemical microenvironment of human body tissues [69]. The advantages of OOC include the following: (1) the product can be obtained quickly, (2) drugs and dosages can be tested simultaneously, (3) the cost of manufacturing is relatively low, (4) because of their small size, OOCs can be portable, and (5) numerous microfluidic systems can be combined onto a single chip, saving both money and space. Therefore, OOCs have been used in many biomedical fields since 2010 [85,86].

The formation of microvascular networks and maintenance of neural activity and function depend on neurovascular interactions. Osaki et al. demonstrated that microvascular and neuronal network models were simultaneously generated from ESC-derived MN spheroids and ECs in microfluidic devices [80]. Studies of the NMJ model are also being actively conducted. Osaki et al. [56] reported a comprehensive protocol for generating hiPSC-derived motor nerve organoids using a microfluidic tissue culture chip. Using this protocol, NMO-containing axon bundles can be obtained faster than using the 2D protocol. Stoklund Dittlau et al. developed a standardized co-culture model using myotubes obtained from the first human primary mesoangioblasts (MAB) and MNs derived from hiPSCs [63]. They established a motor unit system that was comparatively simple to use for investigating the functionality of human NMJs. Furthermore, Yamamoto et al. constructed a microdevice that can precisely compartmentalize the co-culturing of MNs and SkM [64]. The MN spheroids created hiPSCs in one chamber and extended their axons into microtunnels, leading to the formation of functional neuromuscular connections with muscle fibers and tissue-engineered human SkM in the SkM chamber.

The disadvantages of OOC include the following: (1) the volume effect is subordinated to the surface effects as the fluids are extremely small, (2) the necessary fluids may not mix effectively as laminar flow exists where numerous fluids intersect, and (3) special equipment is required to obtain accurate results [86].

### 4.2. Bioprinting

3D bioprinting is the process of employing additive manufacturing to print cells, biocompatible materials, and supporting structures into intricate 3D living tissues with the required cell or organoid architecture and functionality. 3D bioprinting has been applied not only to manufacture functional tissues for transplant purposes, but also to the direct production of 3D-bioprinted tissue models for drug screening and profiling in addition to scaffolds for 3D cell cultures [69]. Moreover, using 3D bioprinting equipment, a very small volume of liquid sample (less than a few nanometers) can be processed with high consistency and reproducibility [77].

A bioengineered skeletal muscle model must be manufactured to closely resemble the structural and functional characteristics of the native in vivo SkM to understand the physiology of skeletal muscle development and the structural and functional characteristics of native in vivo skeletal muscles [60]. Capel et al. developed a scalable model (25–500 μL construct volumes) that allows for the rapid, reliable manufacturing of mature primary human SkM [60]. They provide a method to overcome the limited number of biopsy cells, allowing for the high-throughput screening of functioning human tissues. Culturing MNs in bioengineered muscles is important for understanding the growth and maturation of NMJs. Kim et al. showed that the incorporation of human neural stem cells into 3D bioprinted skeletal muscle constructs strongly implies that human muscle progenitor cells will differentiate and mature more quickly and survive for a longer period and that NMJ creation with AChR clustering in vitro will be successful [87].

However, the 3D bioprinting technology has certain limitations. 3D bioprinting has problems related to printing accuracy and efficiency. The printing efficiency decreases with increasing resolution and finer structures [88]. Another challenge involves addressing a growing number of issues, including the requirements for cells and materials, the maturation and functionality of tissues, and the establishment of proper vascularization and innervation [69].

## 5. NMD Models

### 5.1. Amyotrophic Lateral Sclerosis (ALS)

ALS (Lou Gehrig’s disease) is a neurological disorder that causes progressive paralysis, muscle atrophy, and death as NMJs degenerate. There are two types of ALS: familial and sporadic. One of the causes of the disease is genetic mutations (e.g., superoxide dismutase (*SOD1*), TAR DNA binding protein-43 (*TDP-43*), chromosome 9 open reading frame 72 (*C9orf72*), and *FUS*). Familial ALS is associated with pathogenic variants in *SOD1*. Pathogenic variants in *C9orf72*, *TDP-43*, and *FUS* are associated with familial and sporadic ALS [40,61,67,89]. 

Massih et al. developed an in vitro model that generates NMJs via a co-culture system consisting of iPSC-derived MNs with *SOD1* pathogenic variants and 3D skeletal muscle tissue to study NMJ physiology and dysfunction [61]. This model exhibits features characteristic of ALS, such as decreased muscle contraction and pathological traits. However, the authors mentioned the challenge in validating the developmental stage of bioengineered NMJs due to the difficulty in identifying innervated muscle fibers and corresponding MNs within the co-culture’s size and 3D structure. Osaki et al. developed an ALS-on-a-chip technology (i.e., an ALS motor unit) by co-culturing *TDP-43* pathogenic variants with sporadic ALS patient-iPSC-derived MN spheroids and normal human-derived 3D SkM bundles [67]. In addition, the ALS-on-a-chip model better mimics drug delivery to the central nervous system, including the endothelial cell barrier. Thus, Osaki et al. [67] provided a platform for drug screening and research on ALS pathogenesis. Zhao et al. developed an ALS model by co-culturing iPSC-derived astrocytes (*C9orf72* pathogenic variants) and iPSC-derived MNs. This model demonstrated key symptoms of ALS and functional loss due to specific current reduction. Additionally, removing the *C9orf72* mutation using CRISPR/Cas9 reversed these effects [40]. The authors reported the *C9orf72* mutation’s direct molecular and cellular effects on human astrocytes and their interactions with human MNs. Therefore, this model provides understanding of how the *C9orf72* mutation operates in astrocytes, contributing to the comprehension of non-cell autonomous toxicity in ALS and the development of therapeutic strategies. Stoklund Dittlau et al. also generated *FUS* mutation using CRISPR/Cas9-mediated genome editing and investigated its effects on NMJ formation and function. This model was constructed using iPSC (*FUS* mutation)-derived MNs and mesoangioblast-derived myotubes through a microfluidic co-culture system [63]. In addition, they demonstrated the beneficial effects of tubastatin A (HDAC6 inhibition) as a potential treatment strategy for ALS.

### 5.2. Myasthenia Gravis (MG)

MG is an autoimmune, neuromuscular disorder that causes weakness of fatigable muscles. In 80–85% of MG cases, nicotinic acetylcholine receptor-targeting antibodies cause widespread MG [90,91].

Afshar Bakooshli et al. established MG model with reduced contraction of innervated tissue by treating lgG isolated from MG patients in a co-culture system with myogenic progenitor cells and ESC-derived MNs [23]. They offered a way to investigate MG and personalized drugs in an in vitro 3D co-culture system. Similarly, MG modeling study was also reported to specifically target the NMJ by treating MG patients-derived IgG to NMO [21]. Furthermore, Smith et al. developed the first functional in vitro MG model using a human-on-a-chip derived from human iPSC-MNs and primary SkM [90]. They induced clinically relevant MG pathology to investigate the ability of an antibody against the nAChR α1, a non-specific IgG1 antibody, or human complement sera. In addition to antibodies, complement activation damages the NMJ membrane folds in MG patients, resulting in membrane-attack complexes (MACs). A study investigating the expression of CD59, a MAC inhibitory protein, was reported as a new treatment strategy, but the number of patients in this study was small and there were problems with reproducibility of CD59 and AChr expression using muscle biopsies [91].

### 5.3. Others 

Other rare NMDs include Pompe disease and Limb-girdle muscular dystrophy (LGMD). In Pompe (glycogen storage disease type II) disease, the first in vitro 3D model of human SkM derived from Pompe disease patients was reported. In this model, specific gene expression and physiological characteristics associated with Pompe disease were observed, but no functional damage was observed [57]. Other studies reported the development of a central nervous system model for Pompe disease using neural stem cells derived from patient iPSCs [92]. Among LGMD subtypes, an NMJ model for LGMD2D has been reported. This model was developed through co-culturing of LGMD type 2D patient-iPSC-derived immortalized myoblasts and healthy donor-iPSC-derived MNs using microfluidics and OOC technology [93]. GNE myopathy (GNEM) is caused by pathogenic variants in the gene encoding UDP-N-acetylglucosamine 2-epimerase/N-acetylmannosamine kinase (GNE). GNE proximal degenerative disease models with different mutations using hPSC have been reported [94,95]. To the best of our knowledge, there are no reports on the modeling of human NMJ structure and function in Pompe disease and GNEM.

## 6. Conclusions

We focused primarily on 2D and 3D models of human neuromuscular tissue to highlight the advantages and disadvantages of each approach. As discussed above, all the various 2D and 3D neuromuscular in vitro models developed thus far exhibit both advantages and disadvantages, representing incremental progress from real human neuromuscular tissues in vivo. Therefore, it is important to use appropriate adaptations and controls to ensure the use of optimal in vitro models for each study. With the advancement of technologies that compensate for the shortcomings of each approach, NMO research will continue to develop to better mimic tissues in vivo. This will provide a better understanding of the development of neuromuscular tissue, mechanisms of NMD action, and tools applicable to preclinical studies, including drug screening and toxicity tests.

## Figures and Tables

**Table 1:** Summary of hPSC-derived MN and/or SkM by transgene or small molecules in 2D culture since 2018.

A. Transgene-Based						
Cell Type	Cell Source	Transgene	Function		Disease Modeling	Ref.
			Stimulation	Readout		
MN	hiPSC	NGN2, ISL-1, LHX3 (Tet-on system, PiggyBac)	—	patch-clamp recordings	—	[26]
	hiPSC	NGN2, ISL-1, LHX3 (Lentiviral)	—	—	—	[27]
	hiPSC	NGN2, ISL-1, LHX3, NGN2 + ISL1 + PHOX2A (PiggyBac)	—	patch-clamp recordings	—	[28]
	hiPSC	NGN2, LHX3, ISL1, NeuroD1, ASCL1, MYT1L, BRN2 (Lentiviral)	Glutamate	patch-clamp recordings	Amyotrophic lateral sclerosis/ frontotemporal dementia (ALS/FTD)	[29]
SkM	hiPSC (*CAPN3* pathogenic variants)	Pax7 (lentiviral)	—	—	Limb-girdle muscular dystrophy 2A (LGMD2A)	[30]
	hESC, hiPSC	MyoD (SeV)	EFS	kinetic fluorometric plate reader	—	[31]
	hiPSC	MyoD, BAF60C (piggyBac)	Electrical pacing	xCELLigence^®^ RTCA CardioECR System	Duchenne muscular dystrophy (DMD)	[32]
	hiPSC	MyoD (PiggyBac) SMCHD1 (CRISPR/Cas9 system)	—	—	Facioscapulohumeral muscular dystrophy (FSHD)	[33]
**B. Transgene-Free**						
**Cell Type**	**Cell Source**	**Small Molecule**	**Function**		**Disease** **Modeling**	**Ref.**
			**Stimulation**	**Readout**		
MN	hiPSC	CHIR, SB, Dorsomorphin, RA, PMA, bFGF, hLIF, BDNF, GDNF, dbcAMP NGN2, ISL-1, LHX3 (AdV-Transgenes)	—	—	ALS (TDP-43_CRISPR/Cas-9)	[34]
	hiPSC	RA, PMA, BDNF, GDNF, IGF-1, c-AMP	—	patch-clamp recordings, Ca^2+^ imaging	—	[35]
	hiPSC	LDN, SB, IWR1e, CHIR, RA, PMA, dbcAMP, DAPT	—	MEA, Ca^2+^ imaging	—	[36]
	hiPSC	GDNF, BDNF, IGF-1, CNTF	—	Live imaging	—	[37]
	hiPSC	SB, Dorsomorphin, FGF2, Noggin, RA, SHH, BDNF, GDNF, IGF-1	—	patch-clamp recordings	Charcot–Marie–Tooth disease (CMT) Type II	[38]
	hiPSC	SB, CHIR, Dorsomorphin, Compound E, bFGF, EGF, RA, SHH, PMA, SAG, CNTF, BDNF, NT-3, GNDF	—	Calcium Activity patch-clamp recordings	—	[39]
MN and astrocytes	hiPSC	MN: LDN, FGF, RA, BDNF, GDNF, PMA (sphere culture) Astrocytes: FGF-2, EGF, CNTF	—	Ca^2+^ imaging	ALS (C9orf72_CRISPR/Cas-9)	[40]
SkM	hiPSC	FGF2, CHIR, DAPT, FGF-8, PD, LDN, SB, PMP, XAV, BMP4, RA, TGFβ2, TGFβ3, PMA	—	—	DMD	[41]
	hiPSC	CHIR, BMP4, DAPT, recombinant bFGF, LDN, recombinant IGF-1, recombinant HGF	—	—	DMD	[42]
	hiPSC	CHIR, LDN, hEGF, hFGF-2	spontaneous or 10 mM caffeine	—	ALS	[43]
	hiPSC	CHIR, LDN, SB, FGF-2, EGF, HGF, IGF-1, DAPT	—	—	DMD	[44]
	hiPSC	CHIR, FGF2	—	—	classic infantile Pompe disease	[45]
MN and SkM	hiPSC	ITS-A, LDN, CHIR, IGF-1, HGF, DAPT	spontaneous contraction		DMD DM1 FSHD2 LGMD2A	[46]

**Table 2:** Summary of hPSC-derived MN and/or SkM and NMJ complexes in 3D culture since 2018.

Cell Type	Cell Source	Platform	Function		Disease Modeling	Ref.
			Stimulation	Readout		
MN	hiPSC	Ultra-low attachment plate	—	MEA	—	[55]
	hiPSC	Organoids in microfluidic devices (PDMS)	—	Calcium imaging	—	[56]
SkM	Human biopsy	Cell/hydrogel in PDMS molds	EFS	Contraction	Pompe disease	[57]
	Human biopsy	Cell/hydrogel in PDMS molds	Electrical stimulation	Ca^2+^ transients Contraction	Atrophy, lower contractility and differentiation ability in senescent muscles	[58]
	Human biopsy	3D bioprint (FDM parts- PLA, Collagen/Matrigel^®^ hydrogels)	EFS	Contraction	Regenerate function observed after barium chloride injury	[59]
	Human biopsy	3D bioprint (FDM parts- PLA, LS parts in polyamide-12). Collagen hydrogel, Collagen/Matrigel^®^	Electrical stimulation	Contraction	—	[60]
SkM + MNs	Human biopsy, hiPSC	Microfabrication of the 3D culture dish (PDMS, hydrogel)	Glutamate	Calcium imaging	ALS	[61]
	Human biopsy, hiPSC	3D bioprint (type I collagen/hydrogel)	EFS, spontaneous contraction	—	—	[62]
	hiPSC	Microfluidic devices	—	Calcium fluorescent imaging	ALS (*FUS* mutation)	[63]
	hiPSC	Human neuromuscular tissue-on-a-chip	Glutamate, injected Cell Brite™ membrane dyes	—	—	[64]
	hESC, hiPSC,	Cell/Hydrogel	Electrical and optogenetic stimulation	Ca^2+^ transients contraction	Myasthenia gravis (MG)	[23]
NMJ complex	hiPSC	Non-adherent culture	Optogenetic stimulation	Calcium imaging, patch-clamp recordings	ALS	[65]
	hiPSC	Organoids on low adhesion plates	Optogenetic stimulation	Ca^2+^ transients contraction	—	[66]
	hiPSC	Organoids on low adhesion plates	—	Calcium imaging	MG patient antibodies reduce NMJ function	[21]
	Human biopsy, ESCs	Three compartment microfluidic device	Electrical stimulation	Contraction	ALS	[67]

## Data Availability

Not applicable.

## Abbreviations

MN: motor neuron; SkM, skeletal muscle; hiPSC, human-induced pluripotent stem cell; hESC, human embryonic stem cell; SeV, Sendai virus; AdV, adenovirus vector; EFS, electrical field stimulation; MEA, Multiple electrode array; ALS, amyotrophic lateral sclerosis; FTD, frontotemporal dementia; LGMD2A, limb-girdle muscular dystrophy 2A; FSHD and FSHD2, facioscapulohumeral muscular dystrophy; DMD, Duchenne muscular dystrophy; DM1, myotonic dystrophy; RO, RO4929097; SU, 5402; CHIR, CHIR99021; LDN, LDN193189; SB, SB431542; RA, retinoic acid; PMA, purmorphamine; FGF, fibroblast growth factor; bFGF, basic FGF; hLIF, human leukemia inhibitory factor; IGF-1, insulin growth factor-1; BDNF, brain-derived neurotrophic factor; GDNF, glial cell line-derived neurotrophic factor; CNTF, ciliary neurotrophic factor; dbcAMP, dibutyryl cyclic adenosine monophosphate; SHH, sonic hedgehog; DAPT, N-[N-(3,5-difluorophenacetyl)-l-alanyl]-S-phenylglycine t-butyl ester; EGF, epidermal growth factor; NGF, nerve growth factor; NT-3, Neurotrophin-3; ITS-A, insulin-transferin-selenium-A; MEA, microelectrode array; MG, myasthenia gravis; PDMS, Polydimethylsiloxane; FDM, fused deposition modeling; PLA, polylactic acid; LS, laser sintering.
